# Supplementary material for: A Novel Detection Procedure for Mutations in the 23S rRNA Gene of Macrolide-Resistant Mycoplasma pneumoniae with Two Non-Overlapping Probes Amplification Assay
Source: Microorganisms. 2023 Dec 28;12(1):62. doi: 10.3390/microorganisms12010062 (PMC10820694; doi:10.3390/microorganisms12010062)
Supplement: Supplementary file 1 [file microorganisms-12-00062-s001.zip › microorganisms-2771650-supplementary.pdf]

Supplementary

Table S1. Comparison of the results about drug-resistance testing of *M. pneumoniae* with existing testing methods in 92 clinical specimens.

ID	Sample Type	△CT method				Real-time PCR [a]	Culture [b]	23S rRNA Mutation [c]
		FAM	VIC	△CT	Results			
ATCC29342	Standard Strain	15.92	15.75	0.17	S	Pos	Pos	None
ATCC15531	Standard Strain	16.95	16.51	0.44	S	Pos	Pos	None
ATCC29085	Standard Strain	17.48	17.50	0.02	S	Pos	Pos	None
ATCC29343	Standard Strain	17.64	17.45	0.19	S	Pos	Pos	None
1	Oral Swab	23.65	26.14	2.49	R	Pos	Pos	A2063G
2	Oral Swab	24.94	27.77	2.84	R	Pos	Pos	A2063T
3	Oral Swab	25.04	25.00	0.03	S	Pos	Pos	None
4	Oral Swab	25.10	27.68	2.58	R	Pos	Pos	A2063G
5	Oral Swab	25.42	29.10	3.68	R	Pos	Pos	A2063G
6	Oral Swab	26.25	25.98	0.28	S	Pos	Pos	None
7	Oral Swab	26.60	26.18	0.42	S	Pos	Pos	None
8	Oral Swab	26.89	30.27	3.39	R	Pos	Pos	A2063G
9	Oral Swab	27.68	27.49	0.19	S	Pos	Pos	None
10	Oral Swab	28.88	32.70	3.83	R	Pos	Pos	A2063G
11	Oral Swab	29.01	28.93	0.08	S	Pos	Pos	None
12	Oral Swab	29.13	28.85	0.28	S	Pos	Neg	NT
13	Oral Swab	29.21	33.87	4.66	R	Pos	Pos	A2063G
14	Oral Swab	29.66	32.64	2.99	R	Pos	Pos	A2063G
15	Oral Swab	29.95	33.34	3.39	R	Pos	Pos	A2063G
16	Oral Swab	29.98	32.27	2.28	R	Pos	Neg	NT
17	Oral Swab	30.02	33.65	3.63	R	Pos	Pos	A2063G
18	Oral Swab	30.57	33.85	3.29	R	Pos	Neg	NT
19	Oral Swab	31.03	35.38	4.36	R	Pos	Pos	A2063G
20	Oral Swab	31.04	30.60	0.44	S	Pos	Pos	None
21	Oral Swab	31.07	34.80	3.73	R	Pos	Neg	NT
22	Oral Swab	31.15	34.45	3.30	R	Pos	Neg	NT
23	Oral Swab	31.58	35.97	4.39	R	Pos	Pos	A2063G
24	Oral Swab	31.98	35.72	3.74	R	Pos	Pos	A2064G
25	Oral Swab	32.12	37.85	5.73	R	Pos	Pos	A2063G
26	Oral Swab	32.35	35.46	3.11	R	Pos	Neg	NT
27	Oral Swab	32.60	37.90	5.30	R	Pos	Pos	A2063G
28	Oral Swab	33.34	38.28	4.94	R	Pos	Neg	NT
29	Oral Swab	33.73	37.50	3.76	R	Pos	Pos	A2064G
30	Oral Swab	33.91	38.83	4.92	R	Pos	Pos	A2063G
31	Oral Swab	34.64	39.55	4.90	R	Pos	Pos	A2063G
32	Oral Swab	34.82	40.88	6.06	R	Pos	Pos	A2063G
33	Oral Swab	34.89	40.57	5.68	R	Pos	Neg	NT
34	Oral Swab	35.08	41.01	5.94	R	Pos	Neg	NT
35	Oral Swab	35.09	40.84	5.75	R	Pos	Neg	NT
36	Oral Swab	35.44	40.44	5.01	R	Pos	Pos	A2063T
37	Oral Swab	35.45	40.80	5.35	R	Pos	Neg	NT
38	Oral Swab	35.54	40.42	4.88	R	Pos	Neg	NT

39	Oral Swab	36.21	35.96	0.25	S	Pos	Neg	NT
40	Oral Swab	36.24	35.88	0.36	S	Pos	Neg	NT
41	Oral Swab	36.77	36.81	0.04	S	Pos	Neg	NT
42	Oral Swab	37.02	36.94	0.08	S	Pos	Neg	NT
43	Oral Swab	37.14	37.29	0.16	S	Pos	Neg	NT
44	Oral Swab	37.10	39.94	2.84	R	Pos	Neg	NT
45	Oral Swab	37.70	42.09	4.39	R	Pos	Neg	NT
46	Oral Swab	38.07	37.86	0.21	S	Pos	Neg	NT
47	Oral Swab	38.14	43.44	5.31	R	Neg	Neg	NT
48	Oral Swab	38.44	38.58	0.14	S	Pos	Neg	NT
49	Oral Swab	39.48	39.35	0.13	S	Pos	Neg	NT
50	Oral Swab	39.85	40.00	0.15	S	Pos	Neg	NT
51	Oral Swab	40.68	NT	＞5	R	Pos	Neg	NT
52	Oral Swab	40.87	NT	＞5	R	Pos	Neg	NT
53	Oral Swab	Neg	Neg	Neg	Neg	Neg	Neg	NT
54	Oral Swab	Neg	Neg	Neg	Neg	Neg	Neg	NT
55	Oral Swab	Neg	Neg	Neg	Neg	Pos	Neg	NT
56	Oral Swab	Neg	Neg	Neg	Neg	Neg	Neg	NT
57	Oral Swab	Neg	Neg	Neg	Neg	Neg	Neg	NT
58	Oral Swab	Neg	Neg	Neg	Neg	Neg	Neg	NT
59	Oral Swab	Neg	Neg	Neg	Neg	Neg	Neg	NT
60	Oral Swab	Neg	Neg	Neg	Neg	Neg	Neg	NT
61	Oral Swab	Neg	Neg	Neg	Neg	Neg	Neg	NT
62	Oral Swab	Neg	Neg	Neg	Neg	Pos	Neg	NT
63	Oral Swab	Neg	Neg	Neg	Neg	Neg	Neg	NT
64	Oral Swab	Neg	Neg	Neg	Neg	Neg	Neg	NT
65	Oral Swab	Neg	Neg	Neg	Neg	Pos	Neg	NT
66	Oral Swab	Neg	Neg	Neg	Neg	Neg	Neg	NT
67	Oral Swab	Neg	Neg	Neg	Neg	Neg	Neg	NT
68	Oral Swab	Neg	Neg	Neg	Neg	Neg	Neg	NT
69	BALF	18.25	17.79	0.46	S	Pos	Pos	None
70	BALF	19.18	18.86	0.32	S	Pos	Pos	None
71	BALF	21.07	20.88	0.19	S	Pos	Pos	None
72	BALF	21.11	20.87	0.24	S	Pos	Pos	None
73	BALF	21.87	24.65	2.77	R	Pos	Pos	A2063G
74	BALF	23.63	27.55	3.92	R	Pos	Pos	A2063G
75	BALF	24.45	26.97	2.52	R	Pos	Pos	A2063G
76	BALF	24.79	27.73	2.94	R	Pos	Pos	A2063G
77	BALF	24.89	28.24	3.35	R	Pos	Pos	A2063G
78	BALF	25.01	25.08	0.08	S	Pos	Pos	None
79	BALF	25.21	24.85	0.36	S	Pos	Pos	None
80	BALF	25.60	28.99	3.40	R	Pos	Pos	A2063G
81	BALF	27.22	26.88	0.34	S	Pos	Neg	NT
82	BALF	27.83	32.43	4.60	R	Pos	Pos	A2063G
83	BALF	28.11	32.13	4.02	R	Pos	Pos	A2064G
84	BALF	28.94	32.91	3.98	R	Pos	Pos	A2063G
85	BALF	29.21	29.25	0.04	S	Pos	Pos	None
86	BALF	29.69	34.16	4.47	R	Pos	Pos	A2063G
87	BALF	34.18	38.69	4.51	R	Pos	Neg	NT

88	BALF	Neg	Neg	Neg	Neg	Neg	Neg	NT
89	BALF	Neg	Neg	Neg	Neg	Neg	Neg	NT
90	BALF	Neg	Neg	Neg	Neg	Neg	Neg	NT
91	BALF	Neg	Neg	Neg	Neg	Neg	Neg	NT
92	BALF	Neg	Neg	Neg	Neg	Neg	Neg	NT

Note: S: MSMP positive; R: MRMP positive; Pos: *M. pneumoniae* positive; Neg: *M. pneumoniae* negative; NT: Not detection; None: No nucleic acid mutations detected.

a, b, c: The testing data is sourced from previous publications (References 21,24,25).
